# The influence of expectation on spinal manipulation induced hypoalgesia: An experimental study in normal subjects

**DOI:** 10.1186/1471-2474-9-19

**Published:** 2008-02-11

**Authors:** Joel E Bialosky, Mark D Bishop, Michael E Robinson, Josh A Barabas, Steven Z George

**Affiliations:** 1 University of Florida Department of Physical Therapy, Gainesville, Florida, USA; 2 University of Florida Department of Clinical and Health Psychology, Gainesville, Florida, USA

## Abstract

**Background:**

The mechanisms thorough which spinal manipulative therapy (SMT) exerts clinical effects are not established. A prior study has suggested a dorsal horn modulated effect; however, the role of subject expectation was not considered. The purpose of the current study was to determine the effect of subject expectation on hypoalgesia associated with SMT.

**Methods:**

Sixty healthy subjects agreed to participate and underwent quantitative sensory testing (QST) to their leg and low back. Next, participants were randomly assigned to receive a positive, negative, or neutral expectation instructional set regarding the effects of a specific SMT technique on pain perception. Following the instructional set, all subjects received SMT and underwent repeat QST.

**Results:**

No interaction (p = 0.38) between group assignment and pain response was present in the lower extremity following SMT; however, a main effect (p < 0.01) for hypoalgesia was present. A significant interaction was present between change in pain perception and group assignment in the low back (p = 0.01) with participants receiving a negative expectation instructional set demonstrating significant hyperalgesia (p < 0.01).

**Conclusion:**

The current study replicates prior findings of c- fiber mediated hypoalgesia in the lower extremity following SMT and this occurred regardless of expectation. A significant increase in pain perception occurred following SMT in the low back of participants receiving negative expectation suggesting a potential influence of expectation on SMT induced hypoalgesia in the body area to which the expectation is directed.

## Background

A growing body of evidence supports spinal manipulative therapy (SMT) as an effective treatment for low back pain [1-6]. Furthermore, the evidence is particularly strong when patients are classified into subgroups by patterns suggesting the likelihood of a favorable response [2,3,6]. Despite the positive findings of clinical trials, the mechanisms through which SMT acts are not established.

Hypoalgesia has been associated with SMT and has a postulated involvement in the clinical effectiveness [7-16]. For example, Vicenzino et al [14] observed greater pain free grip and pain pressure threshold in the forearm following SMT to the cervical spine. A prior study by our group found hypoalgesia of c- fiber mediated pain as measured by lessening of temporal summation in the lower extremity following SMT to the lumbar spine [7]. Temporal summation results from multiple painful stimuli of the same intensity applied at a frequency of less than 3 seconds and has been observed in both healthy subjects [17-19] and those experiencing chronic pain [20,21]. Activation of the dorsal horn of the spinal cord has been directly observed with temporal summation in animal studies [22-25]. Subsequently, we interpreted our prior findings of hypoalgesia of temporal summation following SMT in healthy participants as indicative of a pain inhibiting effect occurring at the dorsal horn.

A criticism of prior studies of SMT is a lack of consideration for the influence of non- specific effects such as placebo and expectation [26-28]. The failure to account for non- specific effects may be significant as expectation has demonstrated a robust influence in the general pain literature [29-40]. Specific to manual therapy, Kalauokalani et al [39] report on a secondary analysis of subjects with low back pain who were randomly assigned to receive either acupuncture or massage treatments. Subjects with higher expectations for the effectiveness of their assigned treatments demonstrated greater improvement in function. In our prior study, we attributed hypoalgesia of c- fiber mediated pain in response to SMT to a local spinal cord effect. However, a limitation of our prior study was the failure to account for the potential influence of non- specific effects. Therefore, the purpose of this study was to determine how subjects' expectation about the effect of SMT would influence hypoalgesia. Similar to prior studies [7-15], we expected a hypoalgesic effect in response to SMT, however we hypothesized this effect would be greater in subjects receiving positive expectation regarding the SMT procedure as compared to those receiving neutral or negative expectation.

## Methods

### Subjects

The University Institutional Review Board approved the current study. Subjects were recruited from the University Health Science Center community by flyer and word of mouth. Potential participants were made aware of the methodology of the study and screened for appropriateness by a study representative. Subjects wishing to participate then signed an informed consent form. Inclusion criterion was ages eighteen to sixty and exclusion criteria were non-English speaking, systemic medical conditions (e.g. diabetes, hypertension), current use of psychiatric medication, pregnancy, regular use of prescription medication for management of pain, presently experiencing low back pain, or history of surgery to the low back. We elected to limit the current study to pain free participants for several reasons. First, we were primarily interested in the effect of SMT on a pain protocol known to produce temporal summation. While not as robust as observed in pain conditions [20,21,41], temporal summation is observed in healthy subjects [17,19,42]. Second, we were attempting to both replicate the findings of our prior study in which we used pain free participants [7] and to observe the effect of expectation on those findings. Third, use of healthy subjects eliminated confounding of the hypoalgesic response from clinical pain conditions and pain medications.

### Procedures

Demographic information, psychological questionnaires, baseline expectation for pain, and thermal pain sensitivity measures were collected prior to random assignment of the studied intervention.

#### Thermal Pain Sensitivity

Quantitative sensory testing (QST) was performed using the Medoc Neurosensory Analyzer (TSA- 2001, Ramat Yishai, Israel) with hand- held peltier- element- based stimulator. Participants first underwent a practice session in order to familiarize themselves with the pain testing protocol. Participants then underwent the full QST following a previously established protocol to measure c- fiber mediated pain [43,44]. Briefly, c-fiber mediated pain was assessed on the plantar surface of the non- dominant foot using a train of ten consecutive heat pulses of less than one second duration at an inter- stimulus frequently of .33 Hz (temporal summation). The baseline temperature of each pulse was 35°C and temperature peaked at 51°C. Subjects were asked to rate their delayed (second) pain using a NRS. We used the same protocol to assess pain perception in the low back with the stimulator placed above the posterior superior iliac spine on the non- dominant side; however, in a previous pilot study, we found that subjects were unable to differentiate Aδ and c- fiber mediated pain in the low back [7]. Subsequently, while allowing inferences regarding pain perception following SMT, we are unable to attribute the findings in the low back specifically to c- fiber mediated pain. We chose to investigate both the lower extremity and the low back in this study due to prior studies having noted a region specific influence of non- specific effects. For example, a placebo provided with expectation of relieving experimental pain in the hand has been found to exert an effect localized to that hand, without change in pain perception in the other hand or either foot [45]. Our instructional set and the SMT used in this study were specific to the low back. We felt the region specific nature of the instructional set and the SMT technique might localize the influence of non- specific effects associated with SMT to the low back. Including both anatomical areas in our QST protocol allowed us to test the specificity of our instruction set in our data analysis.

#### Expectation Intervention

Immediately following the practice session, participants were asked to quantify the amount of pain they expected to feel during the QST in both their back and lower extremity using separate numeric rating scales (NRS). These ratings served as the baseline expectation for pain. Following initial QST, participants were randomly assigned to receive a positive, a negative, or a neutral expectation instructional set.

Participants in the positive expectation group were told the SMT; "*is a very effective form of manipulation used to treat low back pain and we expect it to reduce your perception of heat pain*."

Participants in the negative expectation group were told the SMT; "*is an ineffective form of manipulation used to treat low back pain and we expect it to temporarily worsen your perception of heat pain*."

Participants in the neutral expectation group were told the SMT; "*is a form of manipulation used to treat low back pain that has unknown effects on perception of heat pain*."

Additional interactions with study personnel and participants were minimized in order to avoid bias.

After receiving the expectation instructional set, participants were asked to quantify the amount of pain they ***expected ***to feel with the QST following SMT. Specifically, subjects were asked to rate the amount of pain they ***expected ***to feel in their low back, and leg respectively utilizing separate NRSs. We expected subjects receiving a negative expectation instructional set to have lower baseline NRS of pain expectations than the NRS of pain expectation obtained following the instructional set. Conversely, we expected subjects receiving a positive expectation instructional set to have higher baseline NRS of pain expectation than the NRS of pain expectation obtained following the instructional set. Subjects then received SMT to their low back.

#### SMT intervention

The SMT technique was used in our prior study [7] and has been shown to be effective in the treatment of low back pain in subjects meeting a clinical prediction rule [2,6]. We wished to minimize any expectation of SMT resulting from the wording of the consent form. Subjects were shown a picture of the technique and received the following written instruction, "You will lie on your back and the researcher will place you in a standard position that involves twisting. Then, a gentle force will be applied to your lumbar spine by pushing on your pelvis." Similar to the protocols used in prior studies, we performed the technique two times on each side. Similar to our prior study, this was done to all subjects regardless of whether a cavitation was experienced during the procedure. Immediately following the SMT, the same quantitative sensory testing protocol was performed.

### Measures

#### Numeric Rating Scale (NRS)

NRSs were used as a measure of expected and perceived pain. Participants were asked to quantify their experienced and expected pain using a numeric rating scale anchored by "0" (no pain at all) and "100" (worst imaginable pain). The NRS is frequently used as a measure of both clinical and experimental pain and has demonstrated sound psychometric properties in previous studies [46-49].

#### Psychological Questionnaires

Psychological questionnaires with known influences on experimental pain [50-53] were chosen and used to evaluate for post-randomization differences that could affect reporting of experimental pain sensitivity.

#### Pain Catastrophizing Scale (PCS)

The PCS consists of 13 items specific to individual coping styles with pain which are each quantified with a five point ordinal scale. Higher scores indicate greater levels of catastrophizing. The score may be taken as a whole or as individual factors of rumination, helplessness, and magnification. Prior studies have validated the factor structure and found good internal consistency reliability and validity of the PCS [54-57].

#### Fear of Pain Questionnaire-III (FPQ-III)

The FPQ-III [58] consists of 30 items, each scored on a 5-point adjectival scale, which measures fear of normally painful situations. Higher scores indicate greater pain related fear. The FPQ has demonstrated sound psychometric properties in both experimental and clinical pain studies [59-61].

#### Anxiety Visual Analog Scale (VAS)

Anxiety was measured through a 10 cm VAS. Subjects were asked to indicate along the VAS anchored with none and most severe anxiety imaginable the amount of anxiety they were currently feeling regarding the experimental pain task they were about to experience. VASs have been used to measure anxiety in other studies and have demonstrated sound psychometric properties [62-65].

### Data analysis

Descriptive statistics were generated for continuous and categorical measures. Univariate ANOVA was used to assess post-randomization differences in continuous variables of demographic, psychological, and baseline thermal testing measures. Chi- square analysis was used to assess post-randomization differences in categorical demographic variables.

Next, we analyzed the effect of our instructional set on expectation for pain in response to QST following SMT and whether this was influenced by body region. A 2 × 2 × 3 repeated measure ANOVA was used to investigate whether change in pre- instructional set and post- instructional set NRS of expected pain differed by body region, time, and group assignment. Pre and post instructional set measures of expectation for both the low back and lower extremity served as the within subject factors while group assignment was the between subject factor. Post hoc testing was performed as necessary by repeated measure ANOVA models.

Finally, we analyzed the effect of SMT on pain perception and whether this was influenced by instructional set or body region. First, we sought to determine whether a difference existed by body region and time in response to SMT between the different expectation groups. A 2 × 2 × 3 repeated measure ANOVA was used to investigate this question with pre and post SMT pain measures in both the back and the lower extremity as the within subject factors and group assignment as the between subject factor. The dependent variable in this model was response to the c- fiber mediated pain protocol as our previous findings found the effects of SMT to be specific to these fibers in the lower extremity [7]. Post hoc testing was performed as indicated by repeated measure ANOVA models.

## Results

Sixty subjects responded to the recruiting efforts and agreed to participate. No baseline differences were observed in demographic findings, responses to psychological questionnaires, or baseline pain measures (Table 1).

**Table 1:** Descriptive information for the sample

	**Positive**	**Neutral**	**Negative**	**Total Sample**	**p**
**Sex: **Male:	5	4	7	16	0.55
Female:	15	16	13	44	
**Age in years (sd)**	22.95 (2.06)	23.10 (3.49)	23.20 (3.66)	23.08 (3.10)	0.97
**Race: **Caucasian:	18	14	16	48	0.23
African					
American:	1	1	1	3	
Other:	1	5	3	9	
**Education (years (sd))**	16.20 (1.32)	16.21 (1.51)	16.03 (1.26)	16.14 (1.35)	0.89
**History of LBP: **Yes:	5	3	2	10	0.70
No:	14	15	17	46	
Missing:	1	2	1	4	
**Pain Threshold**					
Temperature (sd)	42.42 (5.25)	42.99 (2.89)	41.98 (3.12)	42.46 (3.86)	0.72
NRS Rating (sd)	13.89 (11.72)	19.63 (20.33)	17.40 (18.16)	16.97 (17.01)	0.57
**Baseline Pain Testing**					
Lower Extremity NRS (sd)	34.92 (23.24)	34.01 (28.11)	27.62 (20.93)	32.10 (24.02)	0.59
Low Back NRS (sd)	46.22 (24.08)	52.99 (27.05)	43.86 (26.18)	47.76 (25.64)	0.52
**Psychological Questionnaires**					
PCS (sd)	15.15 (9.15)	17.30 (9.53)	14.75 (8.43)	15.73 (8.96)	0.63
FPQ (sd)	72.25 (13.47)	81.1 (19.43)	76.65 (14.81)	76.67 (16.24)	0.23
Anxiety VAS (sd)	23.20 (17.60)	21.89 (23.96)	24.30 (19.76)	23.15 (20.21)	0.94

Sample demographic, baseline pain perception, and psychological characteristics. Variables were all obtained prior to randomization. Significance set at p ≤ 0.05. Temperature measures are in degrees Celsius. Pain ratings obtained through 0 to 100 numeric rating scale (NRS). PCS = Pain Catastrophizing Scale. FPQ = Fear of Pain Questionnaire. VAS = Visual Analog Scale.

### Effect of instructional set on expected pain

Our original plan was to use pre and post instructional set measures of expectation for pain associated with QST in our analysis of the effect of the instructional set. Following data collection, we were concerned that several factors influenced the integrity of this plan. First, we recruited pain-free subjects that were naïve to the sensory testing protocol that we used. Second, the participant's frames of reference may have been different for the two expectation NRSs. Specifically, the baseline NRS was obtained following a brief practice session in which the participants experienced only four thermal stimuli to their lower extremity. The post instructional set expectation NRS was obtained following ten thermal stimuli to both the low back and lower extremity. Our concern was that our practice session was insufficient to allow a baseline rating of pain expectation for valid comparison to the rating provided following the full QST protocol. We were particularly concerned that the healthy sample in our study would not be adequately able to determine expectation for transient, experimental pain based upon limited exposure. Subsequently, we chose to analyze the effectiveness of our instructional set with pre- SMT average pain ratings of the ten c- fiber mediated thermal stimuli protocol as our baseline rating for comparison to the post instructional set expectation rating. We think the average pain rating of baseline testing better represents each participant's frame of reference for the quantitative sensory testing. Consequently, the average pain rating of the ten pre- SMT c- fiber mediated heat impulses served as the first level within subject factor. The post instructional set NRS in which participants were asked to quantify the amount of pain they expected to experience with quantitative sensory testing following the SMT served as the second level within subject factor. Group assignment served as the between subject factor.

**Figure 1:**
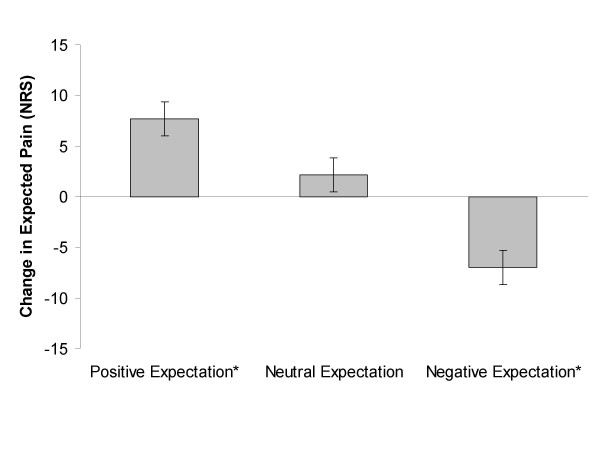
**Effect of Instructional Set on Expected Pain in the Low Back**. Change in expected pain in the low back following instructional set. Positive values indicate expectation of less pain. A statistical interaction occurred with participants receiving a positive expectation instructional set reporting expectations for less pain with quantitative sensory testing (QST) following spinal manipulative therapy (SMT) and those receiving a negative expectation instructional set reporting expectations for greater pain. Error bars represent 1 standard error of the mean (SEM). * indicates significant change at p ≤ 0.05.

A three way interaction was not present between expectation of pain by body area and group assignment (Wilks' Lambda = 0.92, F_(2,53) _= 2.47, p = 0.09, partial η^2 ^= 0.09). A 2 × 3 ANOVA of change in expectation was significant for an interaction in the low back (Wilks' Lambda = 0.85, F_(2,53) _= 4.55, p = 0.02, partial η^2= ^0.15), indicating a differential effect of the instructional set for the low back. Post hoc testing of the low back indicated a significant decrease in expected pain in the positive expectation group (mean difference +7.70, sd = 14.9, p = 0.03, effect size = 0.52), a significant increase in expected pain in the negative expectation group (mean difference -6.98, sd = 15.30, p = 0.05, effect size = 0.46), and no change in the neutral expectation group (mean difference +2.18, sd = 14.91, p = 0.53, effect size = 0.15). (Figure 1) A 2 × 3 ANOVA of change in expectation was not significant for the lower extremity (Wilks' Lambda = 0.95, F_(2,53) _= 1.42, p = 0.25, partial η^2= ^0.05), indicating a main effect of the instructional set for the lower extremity. Pairwise comparison in the lower extremity indicated a mean increase in expected pain of 12.01 (sd = 12.14, p < 0.01, effect size = 0.99).

### Influence of SMT on pain perception in the lower extremity and back by group assignment

A three way interaction existed suggesting change in pain perception differed by body area and group assignment (Wilks' Lambda = 0.88, F_(2,53) _= 3.80, p = 0.03, partial η^2 ^= 0.13).

### Influence of SMT on pain perception in the lower extremity

No interaction between instructional set and pain perception was noted in the lower extremity (Wilks' Lambda = 0.97, F (2,54) = 0.99, p = 0.38, partial η2 = 0.04) suggesting the expectation instructional set did not influence SMT associated hypoalgesia in the lower extremity. A significant main effect (Wilks' Lambda = 0.85, F _(1,54) _= 9.22, p < 0.01, partial η^2 ^= 0.15) was found. Paired t- test determined a mean difference of 4.83 (sd = 12.05) between pre and post SMT pain ratings with post SMT rating being smaller indicating hypoalgesia in the lower extremity after SMT. This difference corresponded to a small effect size of 0.21.

### Influence of SMT on pain perception in the low back by group assignment

**Figure 2:**
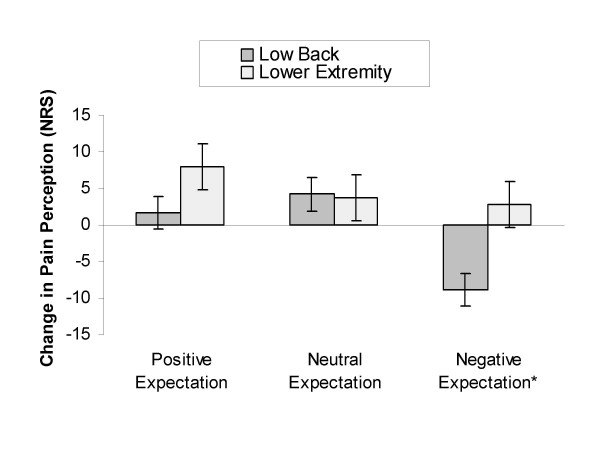
**Change in Pain Perception in the Low Back and Lower Extremity by Expectation Instructional Set**. Change in pain perception in the low back and lower extremity following spinal manipulative therapy (SMT). Positive numbers indicate hypoalgesia, while negative numbers indicate hyperalgesia. A significant interaction was present in the low back suggesting that post SMT pain perception was dependent upon the group to which the participant was randomly assigned. Follow up pairwise comparison indicated a significant increase in pain perception in subjects receiving a negative expectation instructional set. No interaction was observed in the lower extremity of participants; however, a significant main effect occurred suggesting hypoalgesia regardless of group assignment. Error bars represent 1 standard error of the mean (SEM). * indicates a statistically significant change in pain perception in the low back following SMT at p ≤ 0.05.

A significant interaction was present between change in pain perception and group assignment in the low back (Wilks' Lambda = 0.84, F_(2,56) _= 5.35, p = 0.01, partial η^2 ^= 0.16) suggesting a response dependent upon group assignment. Post hoc testing revealed no change in pain perception following SMT in participants receiving the positive expectation instructional set (mean difference +1.66, sd = 13.10, p = 0.57, effect size = 0.13) and the neutral expectation instructional set (mean difference +4.17, sd = 13.10, p = 0.16, effect size = 0.32). Subjects receiving the negative expectation instructional set exhibited a significant increase in pain perception of moderate magnitude following the SMT (mean difference -8.81, sd = 13.42, p < 0.01, effect size = 0.66). (Figure 2)

## Discussion

The current study suggests that hypoalgesia associated with SMT may be influenced by expectation and that this outcome varies by anatomical location in a way that is consistent with the instructional set. Specifically, a hypoalgesic effect was observed in the lower extremity regardless of the provided expectation. Conversely, hyperalgesia was present following SMT in the low back in subjects provided with a negative expectation. This finding is comparable to prior studies which have noted a region specific effect of placebo [45].

A hypoalgesic response to c- fiber mediated pain in the lower extremity of healthy subjects following SMT is consistent with our prior work [7]. Furthermore, the current study suggests that this finding is independent of expectation of pain relief in the low back. Hypoalgesia is suggestive of a mechanism of action of SMT upon neural plasticity. Central sensitization is characterized by perception of pain to previously non- painful stimuli (allodynia) and perception of worsening pain to previously painful stimuli (hyperalgesia). Moreover, central sensitization is hypothesized to be instrumental in the progression of acute to chronic pain and in the maintenance of chronic pain conditions [66]. Subsequently, interventions which inhibit central sensitization may be useful in the prevention of chronic musculoskeletal pain. In fact, others have theorized that the effects of SMT may be due to direct mediation upon the neuroplastic changes which occur with central sensitization [67]. Our repeated finding of c- fiber mediated hypoalgesia in healthy participants suggests that SMT may have a role in the reduction or prevention of neuroplastic changes associated with central sensitization.

Pain perception in the low back following SMT was dependent upon the provided expectation. Specifically, subjects receiving a negative expectation instructional set demonstrated a hyperalgesic response. A role for non- specific effects such as expectation in the outcomes associated with SMT is suggested by this finding. This relationship has not been studied extensively so we have little from the literature with which to compare our findings. However, similar to SMT, acupuncture is an alternative and complementary therapy often used for pain. Non- specific effects have been studied more extensively in the acupuncture literature and this body of work may have applicability to SMT. Functional imaging studies of acupuncture have noted significant overlap in brain activity between actual acupuncture and placebo acupuncture in which subjects believe they are receiving real acupuncture [68]. In contrast, brain activity is dissimilar during placebo acupuncture in which subjects do not believe they are receiving actual treatment [68]. Furthermore, clinical outcomes in acupuncture studies may depend upon the subject's expectations [39,69]. For example, one study noted no difference between actual and placebo acupuncture in analgesic effect; however, subjects who believed they had received the actual acupuncture treatment experienced significantly less pain than those believing they had received the placebo [69]. These studies underscore the significant influence of non- specific effects on clinical outcomes following acupuncture and may have applicability to SMT. Collectively, the findings of our study and the acupuncture literature suggest further investigation into their role in outcomes associated with SMT is warranted.

Limitations of the present study include the use of a healthy sample and a single session of SMT. We do not know individual expectations for pain relief when expectation is not provided and expectation for pain relief from SMT may be different in individuals who seek treatment for musculoskeletal pain. Furthermore, the pre- existing expectations of a sample experiencing clinical pain could be quite different from a healthy sample exposed to transient, experimental pain. Our decision to include a single experimental session rather than multiple sessions is similar to prior studies which have emphasized the immediate hypoalgesic effect of SMT [16], however we acknowledge that the outcomes we observed may differ over repeated sessions. Specifically, patient expectation may change in response to treatment occurring over multiple sessions in the clinical settings, and our methodology did not account for this type of scenario.

Although speculative, and despite these limitations, we think the current study suggests expectation may have a greater influence on pain outcomes in a clinical population for several reasons. First, desire for pain relief has been shown to have a significant effect on placebo analgesia [32,33]. Participants experiencing clinical pain may have greater desire for pain relief with subsequent greater expectations for the benefits of SMT. Our healthy sample may have likely had lower desire for pain relief due to their asymptomatic status and the transient nature of the pain experienced for the purpose of this study. Second, placebo has been shown to have an additive effect and placebo analgesia strengthens with repetition [33]. Consequently, we think that the finding of significant influences of non- specific effects on the outcomes immediately associated with one session of SMT in healthy participants is suggestive of the potential for a much larger effect in individuals seeking treatment for musculoskeletal pain that occurs over multiple sessions. Finally, placebo analgesia is strengthened by expectation. In fact, the magnitude of the placebo effect is heightened in experimental pain studies if the painful stimulus is surreptitiously reduced immediately following the suggestion of pain relief [35]. The influence of expectation on the outcomes associated with SMT may strengthen over multiple sessions as immediate hypoalgesia during an individual session may enhance the effect of expectation over multiple sessions. Future studies of non- specific effects of SMT may wish to account for desire for pain relief and include multiple sessions in order to observe immediate changes in pain perception and their affect on subsequent sessions.

A further limitation of the current study is the lack of a control group not receiving SMT. Such a study design would allow the calculation of the magnitude of the effect in comparison to natural history rather than to other groups receiving SMT. However, we feel our study design is sound for two reasons. First, we controlled for expectation by direct manipulation in both a positive and negative direction. Second, a true control group would be difficult in this type of study, as participants would enter with preconceived expectations. Subsequently, a study design in which a control group was included would require measurement of pre- existing expectation and then controlling for these statistically. As a result, we do not think that the lack of a control group invalidates the findings of the present study.

Despite these limitations, we feel the current study provides preliminary support for the influence of non- specific effects on the outcomes associated with a single session of SMT in normal subjects and that this finding is worthy of further investigation over longer duration and in clinical settings.

## Conclusion

This study provides preliminary evidence for the influence of a non- specific effect (expectation) on the hypoalgesia associated with a single session of SMT in normal subjects. We replicated our previous findings of hypoalgesia in the lower extremity associated with SMT to the low back. Additionally, the resultant hypoalgesia in the lower extremity was independent of an expectation instructional set directed at the low back. Conversely, participants receiving a negative expectation instructional set demonstrated hyperalgesia in the low back following SMT which was not observed in those receiving a positive or neutral instructional set.

## Competing interests

The author(s) declare that they have no competing interests.

## Authors' contributions

All authors read, edited, and approved the final version of the manuscript.

JEB was responsible for the initial conception of the research question, supervising the protocol, data analysis, and manuscript preparation. SZG was responsible for the initial conception of the research question, modifying the research question, supervising the protocol, securing funding, and critically reviewing earlier versions of the manuscript. MDB was responsible for the initial conception of the research question, modifying the research question, and critically reviewing earlier versions of the manuscript. MER was responsible for modifying the research question and critically reviewing earlier versions of the manuscript. JAB was responsible for administering the protocol and critically reviewing earlier versions of the manuscript.

## Pre-publication history

The pre-publication history for this paper can be accessed here:


